# Epidemiologic Profile of Type-Specific Human Papillomavirus Infection after Initiation of HPV Vaccination

**DOI:** 10.3390/vaccines8030425

**Published:** 2020-07-29

**Authors:** Masayuki Sekine, Manako Yamaguchi, Risa Kudo, Sharon J. B. Hanley, Megumi Hara, Sosuke Adachi, Yutaka Ueda, Etsuko Miyagi, Sayaka Ikeda, Asami Yagi, Takayuki Enomoto

**Affiliations:** 1 Department of Obstetrics and Gynecology, Niigata University Graduate School of Medical and Dental Sciences 1-757 Asahimachi-dori, Chuo-ward, Niigata 951-8510, Japan; manako0131@gmail.com (M.Y.); pearpear@med.niigata-u.ac.jp (R.K.); sadachi@med.niigata-u.ac.jp (S.A.); enomoto@med.niigata-u.ac.jp (T.E.); 2 Department of Obstetrics and Gynecology, Hokkaido University Graduate School of Medicine, Sapporo 060-8638, Japan; sjbh1810@mta.biglobe.ne.jp; 3 Department of Preventive Medicine, Faculty of Medicine, Saga University, Saga 849-8501, Japan; harameg@cc.saga-u.ac.jp; 4 Departments of Obstetrics and Gynecology, Osaka University Graduate School of Medicine, Osaka 565-0871, Japan; y.ueda@gyne.med.osaka-u.ac.jp (Y.U.); a.yagi@gyne.med.osaka-u.ac.jp (A.Y.); 5 Department of Obstetrics and Gynecology, Yokohama City University School of Medicine, Yokohama 236-0004, Japan; emiyagi@yokohama-cu.ac.jp; 6 Division of Environmental Medicine and Population Sciences, Department of Social and Environmental Medicine, Graduate School of Medicine, Osaka University, Osaka 565-0871, Japan; sayakaikeda0201@gmail.com

**Keywords:** HPV vaccine, HPV infection, cross-protection, type replacement

## Abstract

Organized human papillomavirus vaccination (OHPV) in Japan was introduced in 2010 for girls aged 12–16 years who were born in 1994 or later. The rate of OHPV coverage was 70–80%. However, after suspension of the government vaccination recommendation, the coverage dramatically decreased. We aim to investigate the change in prevalence of HPV infection after the initiation of HPV vaccination. We recruited females aged 20–21 years attending public cervical cancer screening from 2014 to 2017 fiscal years (April 2014 to March 2018). Residual Pap test specimens were collected for HPV testing. We compared the prevalence of HPV type-specific infection between women registered in 2014 (born in 1993–1994, including the pre-OHPV generation) and registered in 2015–2017 (born in 1994–1997, the OHPV generation). We collected 2379 specimens. The vaccination coverage figures were 30.7%, 86.6%, 88.4% and 93.7% (*p* < 0.01) from 2014 to 2017, respectively. The prevalence of HPV16/18 infection significantly decreased from 1.3% in 2014 to 0% in 2017 (*p* = 0.02). The three most prevalent types were HPV52, 16 and 56 in 2014, and HPV52, 58 and 56 in 2015–2017, respectively. HPV16 and 33 infection rates decreased. On the other hand, the HPV58 infection rate was obviously increased after OHPV from 0.3% to 2.1%. Our study demonstrates that the prevalence of HPV16/18 infection dramatically decreased and the profile of type-specific HPV infection was changed after OHPV.

## 1. Introduction

In Japan, there are over 10,000 cases of cervical cancer annually; nearly 3000 women die from the disease [1] and the incidence of cervical cancer and high-grade cervical intraepithelial neoplasia has been increasing in young women, especially those in their 20s and 30s [2]. For cervical cancer prevention, as secondary prevention, cancer screening by Pap smear is recommended for women aged 20 years or older every two years. As the primary prevention, an HPV vaccine was approved in 2009, and organized human papillomavirus vaccination (OHPV) in Japan was introduced in 2010 for girls aged 12–16 years who were born in 1994 or later (ethical code 2015–1866). Since April 2013, the HPV vaccine has been part of a national immunization program (NIP), and the rate of OHPV coverage was 70–80% in girls born between 1994 and 1998. However, sensational reports of so-called adverse events were published by the media and rapidly became widespread. As a result, the Japanese government decided to suspend the proactive recommendation of HPV vaccination in June 2013. After this decision, HPV vaccination coverage dramatically dropped to nearly zero, and this remains the situation [3,4]. The girls born in 1994 (OHPV generation) became 20 years old in 2014 and have been included in a public cervical cancer screening. Therefore, it was necessary that we investigate the change in prevalence of HPV infection after the initiation of HPV vaccination in Japanese girls.

## 2. Material and Methods

We recruited 2379 women aged 20–21 years attending a public cervical cancer screening held in Niigata, Japan, from fiscal years (FY) 2014 to 2017 (FY2014–2017: from April 2014 to March 2018). We performed an HPV test, HCII, for screening and used the MEBGEN kit for HPV genotyping. Residual liquid-based cytology (SurePath™ BD Diagnostics, Sparks, MD, USA) specimens from the cervical screening were used for HPV testing. All samples were tested with Hybrid Capture (HC) 2^TM^ (Qiagen, Hilden, Germany) for pooled infection with one or more from 13 high-risk HPV genotypes: HPV types 16, 18, 31,33, 35, 39, 45, 51, 52, 56, 58, 59 and 68. Only those samples positive for the HC2^TM^ test underwent HPV genotyping with the MEBGEN^TM^ HPV kit (MBL, Nagoya, Japan) [5]. In addition, we asked the subjects about their vaccination status and previous sexual behavior in questionnaires. For accuracy, we used official vaccination records to ascertain vaccination status, as well as date of vaccination, type of vaccine administered, and the number of doses administered from municipal records. Of those 2379 participants, immunization status was confirmed by municipal records in 1755 women, and of these, 1709 (97.4%) and 46 (2.6%) women had received the bivalent and quadrivalent vaccine, respectively. Information about sexual history was obtained through a self-administered questionnaire that asked about age at sexual debut and number of previous sexual partners. For the latter, participants had to choose from the following 5 categories: none, 1, 2–5, 6–9 and ≥10. Age at first intercourse was categorized into four groups: 14 years or younger, 15–16 years, 17–19 years and ≥20 years.

The target group in this study is shown in Table 1. The women born in FY1993 (April 1993–March 1994) were the pre-OHPV generation. The women born after FY1994 were eligible for free HPV vaccination (OHPV generation). We compared the prevalence of HPV type-specific infection between FY2014 and FY2015–2017, that is, women registered in FY2014 (born in FY1993–1994) and registered in FY2015–2017 (born in FY1994–1997). The vaccination coverage in the OHPV generation was over 70% [6].

All experiments were performed in accordance with the relevant guidelines and regulations, and written informed consent was obtained from all participants. The present study protocol was approved by the institutional review board of Niigata University Graduate School of Medical and Dental Science and registered at the UMIN Clinical Trials Registry, trial number UMIN000026757. Data were analyzed using EZR (Easy R: Saitama Medical Center, Jichi Medical University, Saitama, Japan), which is a graphical user interface for R (The R Foundation for Statistical Computing, Vienna, Austria). A two-sided *p* value of <0.05 was considered to indicate statistical significance.

## 3. Results

### 3.1. Background of Participants

The background of participants in this study is shown in Table 2. The HPV vaccination rate was 30.7% in 2014, 86.6% in 2015, 88.4% in 2016 and 93.7% in 2017, respectively. Age at sexual debut and number of sexual partners were not statistically different between each fiscal year.

### 3.2. Prevalence of HPV Infection Rate

**Figure 1:**
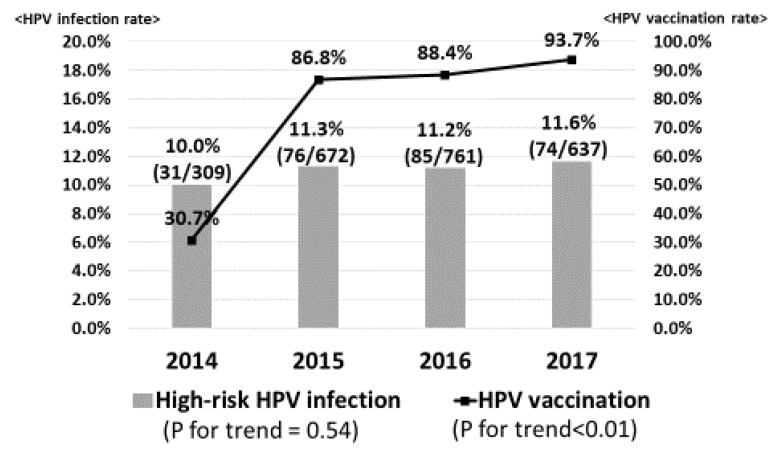
High-risk human papillomavirus (HPV) infection. The HPV vaccination rate was significantly increased from 28.6% in 2014 to 80.0% in 2017. However, the prevalence of high-risk HPV infection was slightly increased from 10.4% in 2014 to 12.4% in 2017.

**Figure 2:**
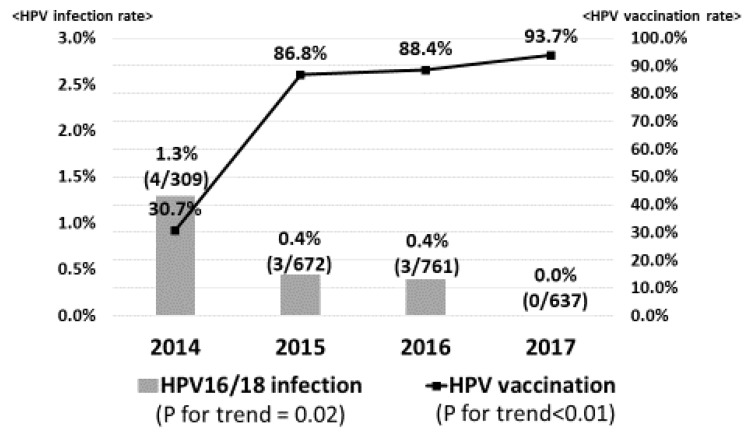
Prevalence of vaccine types HPV16/18 infection. As the HPV vaccination rate increased, the prevalence of vaccine types HPV16/18 infection significantly decreased from 1.3% in 2014 to 0.5% in 2015, 0.4% in 2016 and 0% in 2017, sequentially.

Figure 1 demonstrates the prevalence of high-risk HPV infection. Gradually, the HPV vaccination rate was significantly increased from 30.7% in 2014 to 93.7% in 2017 (*p* for trend <0.01). However, the prevalence of high-risk HPV infection was slightly increased from 10.0% in 2014 to 11.3% in 2015, 11.2% in 2016 and 11.6% in 2017 (*p* for trend = 0.54). On the other hand, in regard to vaccine types HPV16/18 infection, the prevalence significantly decreased from 1.3% in 2014 to 0.4% in 2015, 0.4% in 2016 and 0.0% in 2017 (*p* for trend = 0.02), sequentially (Figure 2).

### 3.3. Profile of Type-Specific HPV Infection

Table 3 shows the prevalent types of HPV infection in each fiscal year. We compared the HPV types between FY2014 (including the pre-OHPV generation) and FY2015–2017 (OHPV generation). The three most prevalent types were HPV52, 16 and 56 in 2014, and HPV52, 58 and 56 in 2015–2017, respectively. HPV16 and 33 infection rates decreased after OHPV from 1.29% to 0.24%, and from 0.65% to 0.34%, respectively. On the other hand, HPV18, 31, 45 and 52 infection rates in 2014 were almost the same as those in 2015–2017. The HPV58 infection rate was obviously increased after OHPV from 0.65% in 2014 to 2.17% in 2015–2017.

## 4. Discussion

In this study, we revealed that the prevalence of high-risk HPV infection was slightly increased after OHPV; nevertheless, the prevalence of vaccine types HPV16/18 infection significantly decreased from 1.3% to 0%. In Japan, 40–50% of cervical cancer is caused by HPV16 infection and 20–30% by HPV18 infection, followed by HPV52/58/31/33 [7,8]. In short, 60 to 70% of cervical cancer is caused by HPV types 16/18 infection, which is slightly lower than that in Western countries. Since HPV16/18 infection has a short period from infection to cancer development [9,10,11], HPV16/18 infection is more common in young patients with cervical cancer. Approximately 90% of cervical cancer patients in their 20s have HPV16/18 infection [12], and it is important to give a vaccine targeting HPV16/18 before sexual intercourse for girls. The bivalent and quadrivalent vaccines approved in Japan are highly effective for HPV16/18 infection, which causes the majority of HPV-related cancers [13,14].

Several studies have reported a “herd immunity” that reduces the HPV16/18 infection rate in unvaccinated people in vaccinated generations in Australia, the United Kingdom, and the United States [15,16,17]. In these countries, the younger generation as a whole receives the benefits of herd immunity from the introduction and spread of the HPV vaccine. In our study, HPV16/18 infection rates declined to 0% in FY2017 (April 2017–March 2018), when vaccination rates increased to 80% after the introduction of NIP. From the result, it was expected that herd immunity would be acquired in Japan in the near future. However, the current vaccination rate is almost 0, and this effect has remained a blueprint and never carried out. If the current situation continues in Japan, there is concern that disparities might occur in the incidence of HPV infections and result in future highly preventable cervical and other HPV-related cancers, depending on an unfortunate year of birth: women born after the year 2000 [18]. It is imperative that the Japanese government resumes proactive recommendations for the HPV vaccine.

The bivalent and quadrivalent vaccines provide cross-protection, predominantly against HPV31/33/45 [13,14,19,20,21,22]. There are some reports that the profile of type-specific HPV infection has changed in women in the vaccination generation; however, there is no such report in Japan so far. According to the spread of the vaccine, as the vaccine types HPV16/18 infection rate decreased, other HPV type infection rates (e.g., HPV35, 39, 51, 52 and 58) relatively increased [23,24]. Our results indicate that there was an increased incidence of HPV51, 58 and 59 types between 2014 and 2015–2017. However, there was a slight increase in HPV35 and 39. In our previous study, we reported that the bivalent vaccine had a significant cross-protection against HPV 31/45/52 infections in Japanese women [25]. Among the cross-protection types 31/45/52, infection rates of all types did not decrease after OHPV in the current study. Regarding the HPV52/58 types, which have the second highest carcinogenic risk after the HPV16/18 type, our results show an increase in the infection rate of HPV58 and almost the same rate of HPV52 between 2014 and 2015–2017. Looking at the profiles of the HPV infection rate in 2015–2017, the top seven types, HPV52, 58, 56, 51, 59, 39 and 68, are neither target nor cross-protection types of the bivalent and quadrivalent vaccines: so-called non-vaccine-type HPV.

“HPV genotype replacement” in HPV vaccination leads to an increase in non-vaccine-type HPV in contrast to the vaccine-targeted and cross-protection types. Several epidemiological data after the initiation of HPV vaccination have shown a slight increase in the pooled prevalence of high-risk non-vaccine-type HPV, not including HPV31/33/45 [20,23]. However, type-specific trends of non-vaccine-type HPV are more heterogeneous without consistent signals of type replacement [26]. The likelihood of type replacement remains ambiguous, and further studies are needed to monitor for cross-protection and possible type replacement after the introduction of the HPV vaccine [23,27,28,29].

The Japanese government approved the manufacturing and marketing of the nonavalent vaccine on 20 May 2020. After approval by the government, further discussions will be conducted in the subcommittee for use in NIP. The nonavalent HPV vaccine may be able to prevent approximately 90% of all HPV-related cervical cancer cases [30] and is a promising vaccine for a country like Japan, where the involvement of high-risk non-16/18 type HPV infections in cervical cancer development is higher than that in Western countries.

This study has several limitations. Firstly, our results may not reflect the population of Japan as a whole, since our study was only performed in one region of Japan. A second limitation is that the HPV vaccination rate was relatively high (30.7%) in 2014, because the group included women born in 1994 (OHPV generation). If the rate had been sufficiently low, the profile of type-specific HPV infection might have clearly changed after initiation of HPV vaccination.

## 5. Conclusions

The prevalence of vaccine types HPV16/18 infection dramatically decreased after the initiation of HPV vaccination. However, the HPV vaccination rate has been nearly 0% in Japan, so Japanese girls are once again at risk for HPV16/18-related cervical cancer. This risk could be mitigated if the Japanese Ministry of Health, Labor and Welfare resumed proactive recommendations for the vaccine.

## Figures and Tables

**Table 1:** Target group in this study.

Fiscal Year	20 Years	21 Years	(Age)
2014	1994	1993	(Birth year)
2015	1995	1994	(Birth year)
2016	1996	1995	(Birth year)
2017	1997	1996	(Birth year)
		1993: pre-OHPV generation	
		1994–1997: OHPV generation	

**Table 2:** Characteristics of participants.

	2014		2015		2016		2017		*p*-Value
	*n* = 309		*n* = 672		*n* = 761		*n* = 637		
HPV Vaccination	95	(30.7%)	583	(86.6%)	673	(88.4%)	597	(93.7%)	<0.01 *
Age at sexual debut	17.5	(±1.9)	17.4	(±1.9)	17.3	(±1.8)	17.5	(±1.8)	0.27 ^†^
Number of sexual partners									
≥10	24	(7.8%)	36	(5.4%)	49	(6.4%)	32	(5.0%)	0.08 *
6–9	15	(4.9%)	50	(7.4%)	46	(6.0%)	26	(4.1%)	
2–5	118	(38.2%)	244	(36.3%)	251	(33.0%)	206	(32.3%)	
0–1	141	(45.6%)	283	(42.1%)	352	(46.3%)	308	(48.4%)	

* Chi-square test; ^†^ ANOVA.

**Table 3:** Prevalent types of HPV infection.

2014 (*n* = 309)			2015–2017 (*n* = 2070)		
Genotyping	*n*	%	Genotyping	*n*	%
HPV52	10	3.24%	HPV52	64	3.09%
HPV16	4	1.29%	HPV58	45	2.17%
HPV56	4	1.29%	HPV56	41	1.98%
HPV33	2	0.65%	HPV51	30	1.45%
HPV51	2	0.65%	HPV59	28	1.35%
HPV58	2	0.65%	HPV39	20	0.97%
HPV68	2	0.65%	HPV68	15	0.72%
HPV31	1	0.32%	HPV31	7	0.34%
HPV39	1	0.32%	HPV33	7	0.34%
HPV59	1	0.32%	HPV35	6	0.29%
HPV18	0	0.00%	HPV16	5	0.24%
HPV35	0	0.00%	HPV45	3	0.14%
HPV45	0	0.00%	HPV18	1	0.05%
